# Improving health and wellness in medically underserved communities: insights, innovations, and applications

**DOI:** 10.1186/s12913-017-2751-9

**Published:** 2017-12-13

**Authors:** Stephen J. O’Connor

**Affiliations:** Department of Health Services Administration, School of Health Professions, University of Alabama at Birmingham, SHP Building 557, 1720 2nd Avenue South, Birmingham, AL 35294 USA

The American health system is deeply rooted in notions of individual freedom, capitalism, and western Judeo-Christian values. Since its founding, the United States has been a free society built on laws, customs, and culturally engrained beliefs that strongly support individual freedoms for its citizens. These freedoms have also extended to an individual’s health. Benjamin Franklin famously remarked that “health is wealth” and observed that health is something an individual is free to improve, conserve, or waste.

Market justice principles propose that healthcare is best distributed in a free economy through market forces. These principles are also the foundation of the capitalist paradigm – where goods and services are efficiently distributed through society, going to those who value them most and who have the ability to purchase them through their own resources – resources obtained through their own efforts. Most goods and services in the U.S. are distributed according to this principle, allowing for efficient product distribution, pricing, quality, and innovation to meet consumer preferences. Thus, there are positive elements associated with the market justice principles embodied within the capitalist paradigm, and it can work well for people who are able to participate in that market through their ability to pay. However, the market justice principle posits “… that giving people something they have not earned would be morally and economically wrong” [1], p. 48. It is this last point that can become problematic when it comes to social goods such as healthcare, and to which we next turn our attention.

Healthcare has been described as a particularly valuable social good [2] and the market justice model, which is deeply engrained in the American psyche, does not work as well for social goods like healthcare. The Benjamin West painting Christ Healing the Sick in the Temple is located in Philadelphia’s Pennsylvania Hospital, which is America’s first hospital. The painting is an artifact of American cultural views of health and healthcare that reflects social justice, healing, and compassion. It also implies an emphasis on individual personal health services over population and public health efforts [3]. Social justice principles see healthcare as a social good, not an economic good, and accordingly is at odds with the tenets of market justice and the capitalist paradigm. Social justice views the resources required for health services delivery to be distributed according to one’s needs, not ability to pay. The long American healthcare debate and continuous reform efforts really boil down to the conflict between market justice and social justice principles. Some stakeholders believe more strongly in one than the other. But as the nation ascribes to both market justice (e.g., free capitalism) and social justice (e.g., western Judeo-Christian values) principles, a workable approach to improving health, wellness, and healthcare access at the national and local levels should draw on the best of those principles.

Through the innovations described in this supplement of BMC Health Services Research, James K. Elrod and John L. Fortenberry, Jr. show how the Willis-Knighton Health System of Shreveport, Louisiana has been able to make steady progress in improving the health and wellness of underserved communities. The first article, “Advancing indigent healthcare services through adaptive reuse: repurposing abandoned buildings as medical clinics for disadvantaged populations,” describes how the acquisition and repurposing of empty structures can be a cost-effective way for providing needed healthcare services to underserved, indigent communities.

Adaptive reuse of historic buildings, for example, is a way to breathe contemporary, purposeful life into old structures while preserving the heritage and character of the communities in which they are located. Such projects, when done carefully, can infuse tired, run down, and vacant areas with a new vitality and attractiveness. The HealthSouth Rehabilitation Hospital of Western Massachusetts [4] and the Vanderbilt University Medical Center, One Hundred Oaks Mall [5] are examples of adaptive reuse of historic buildings for delivery of healthcare services.

Elrod and Fortenberry discuss adaptive reuse of abandoned structures as a unique and integral part of Willis-Knighton’s approach to their facility needs. A 4-step protocol for evaluating potential reuse candidates, known as the Adaptive Reuse Consideration Framework is presented, using Willis-Knighton Health System’s Project NeighborHealth as an illustrative example. This initiative provides an essential health services lifeline to citizens living in areas of extreme poverty who would not otherwise have access to essential healthcare services.

The second article, “Tithing programs: pathways for enhancing and improving the health status of the underprivileged,” describes a program developed at Willis-Knighton Health System known as “Tithing the Bottom Line.” This initiative differs from other healthcare tithing programs such as 1) the Atlantic Stewardship Bank Tithing Program which provides financial support to several categories of recipients, including healthcare facilities [6], and 2) “healthcare sharing ministries” where members who agree to live according to Christian values and attend church services, contribute monthly sharing payments in exchange for having their medical bills paid for by the ministry should the need arise [7].

The Willis-Knighton tithing program takes 10% of earnings before interest, taxes, depreciation, and amortization (EBITDA) expenses and disburses those monies to charitable health system and community organizations providing services deemed as contributing to the health system’s mission and community betterment. High priority activities are those that enhance health and wellness services for disadvantaged, impoverished populations. The authors offer operational guidance to those healthcare organizations seeking to begin their own tithing program.

The third article, “Billboard advertising: an avenue for communicating health information and opportunities to disadvantaged populations,” describes how billboards can be an effective vehicle for healthcare advertising and social marketing. Although billboards have been criticized for the potential visual ugliness they can inflict on open-space landscapes [8], healthcare marketers see billboards as cost-effective tools for communicating their messages and as platforms for promoting more important health-related content. Elrod and Fortenberry show how billboards successfully convey targeted health and wellness information to disadvantaged populations as a social marketing tool that can lead to healthier and improved communities.

The fourth article, “The hub-and-spoke organization design revisited: a lifeline for rural hospitals,” describes the hub-and-spoke design configuration and how this networked structure is supportive of fruitful affiliations with rural hospitals and the healthcare needs of the communities in which these hospitals are located. The environments of rural hospitals in the U.S., unfortunately, tend to be harsh and unforgiving places [9]. This has resulted in financially strained rural hospitals that face significant hurdles in adequately serving their patients with many of these hospitals closing. Under these circumstances, not only does the hospital suffer but so do the communities that are dependent on them. The hub-and-spoke design has been identified as a model for successfully delivering healthcare services in rural and remote areas of the world [10, 11]. Elrod and Fortenberry show how the hub-and-spoke design, through strengthened partnerships resulting from careful implementation, can improve the odds of a rural hospital’s survival and success.

The final article in the supplement, “Bridging access gaps experienced by the underserved: the need for healthcare providers to look within for answers,” describes how the hardships experienced by indigent populations are frequently overlooked. In a capitalist society there are often subsets of people who are unable to participate fully in the economy of that society (e.g., jobs, income, housing, health insurance, etc.) and unable to reap the benefits that participation affords – benefits that include health, wellness, and access to health services. Organizations, such as the Willis-Knighton Health System, with altruistic missions that demonstrate commitment to their communities can develop effective ways to meaningfully serve their marginalized populations. Being close to this population, understanding their needs, and engaging in the hard thinking necessary for dealing with this “wicked problem” can yield beneficial, innovative approaches and results. The strategies and innovations described here can serve as both a guide and an inspiration to other health systems as they seek to meet the broad health and healthcare needs of their entire communities.
